# Calciphylaxis with extensive arterial calcification

**DOI:** 10.1002/ccr3.1068

**Published:** 2017-07-03

**Authors:** Syed Irfan Qadri, Abhilash Koratala

**Affiliations:** ^1^ Division of Nephrology, Hypertension and Renal Transplantation University of Florida Gainesville Florida

**Keywords:** Arterial calcification, calciphylaxis, end‐stage renal disease, hyperphosphatemia

## Abstract

Calciphylaxis is a clinical syndrome of arteriolar calcification with resultant tissue necrosis, most commonly seen in patients with end‐stage renal disease (ESRD) on dialysis. This condition carries a high mortality rate mainly due to infection. Helpful interventions include adequate dialytic therapy, medical management of hyperparathyroidism, hyperphosphatemia, and intravenous sodium thiosulfate therapy.

## Case Description

**Figure 1:**
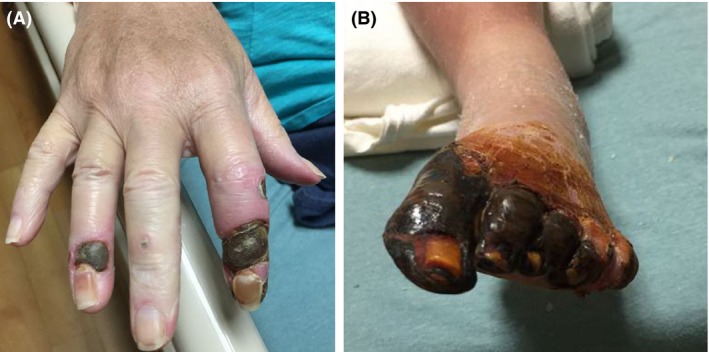
Necrotic lesions surrounded by erythema on the fingers (A) and toes (B) of the right hand and left foot, respectively.

**Figure 2:**
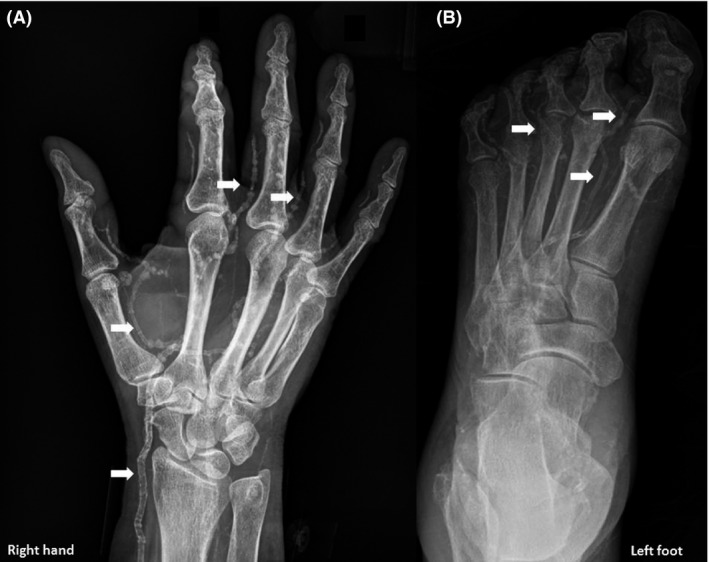
X‐rays of the right hand (A) and left foot (B) showing extensive arterial calcifications, indicated by arrows.

A 54‐year‐old woman with obesity, diabetes mellitus, and ESRD on hemodialysis presented with exquisitely painful lesions on her extremities. Examination revealed necrotic lesions at the tips of right index and ring fingers and left toes, with surrounding inflammation (Fig. 1). There was no obvious infection. Laboratories demonstrated serum calcium of 8.3 mg/dL (8.4–10.2), albumin 3.0 g/dL (3.5–5.0), phosphorus 7.7 mg/dL (2.7–4.5), and parathyroid hormone (PTH) 348 pg/mL (150–300 for ESRD). Her PTH was apparently “very high” several months ago. X‐rays of the hand and foot revealed extensive arterial calcifications suggestive of calciphylaxis (Fig. 2). Risk factors for calciphylaxis include high calcium‐phosphate product, elevated PTH, hypoalbuminemia, diabetes, obesity, warfarin use, female sex, and protein C or S deficiency. Most lesions occur in legs though uncommon sites such as breast have been reported 1. It has been proposed that calciphylaxis and vascular calcification are a continuum of extra‐skeletal osteogenesis and the clinical manifestations depend upon the location of the affected artery 2.Interestingly, medial arterial calcifications are thought to exhibit a linear or railroad‐track arrangement on plain radiographs as in our case, as opposed to patchy intimal calcifications 3. She was treated with sodium thiosulfate and intensification of dialysis regimen, which resulted in some improvement of the lesions.

## Informed Consent

Informed consent has been obtained for the publication of this clinical image.

## Conflict of Interest

The authors have declared that no conflict of interest exists.

## Authorship

Both the authors made substantial contribution to the preparation of this manuscript and approved the final version for submission. SIQ: acquired the images and drafted the initial version of the manuscript. AK: revised the manuscript for critically important intellectual content and approved for final submission.
